# Single Nucleotide Variants in the TLR1, TLR2 and TLR6 Genes: A Case–Control Study in a Colombian Population

**DOI:** 10.3390/tropicalmed8100473

**Published:** 2023-10-16

**Authors:** Luz D. Gutierrez-Castañeda, Carmen R. Acosta, Mónica A. Bustos, Diana K. García, Diana P. Bohada, Raúl Rodríguez, Martha Inirida Guerrero

**Affiliations:** 1 Grupo de Ciencias Básicas en Salud (CBS)-FUCS, Instituto de Investigación, Fundación Universitaria de Ciencias de la Salud-FUCS, Bogotá 111411, Colombia; 2 Grupo Dermatología General, Hospital Universitario Centro Dermatológico Federico Lleras Acosta E.S.E, Bogotá 111511, Colombia; dkgarcia@fucsalud.edu.co; 3 Grupo Dermatología Tropical, Hospital Universitario Centro Dermatológico Federico Lleras Acosta E.S.E, Bogotá 111511, Colombia; carmen.acostapuj@gmail.com; 4 Grupo de Investigación en Enfermedades Parasitarias, Tropicales e Infecciosas (GIEPATI) Universidad de Pamplona, Pamplona 543058, Colombia; monica.bustosmo@unipamplona.edu.co (M.A.B.); dpbohada@unipamplona.edu.co (D.P.B.); rrodriguez@unipamplona.edu.co (R.R.)

**Keywords:** TLR1, TLR2, TLR6, leprosy, single nucleotide variant

## Abstract

Background: Single nucleotide variants in toll-like receptor genes play a crucial role in leprosy susceptibility or resistance. Methods: With an epidemiology case–control study, associations between SNVs rs5743618 in TLR1, rs5743708 in TLR2, and rs5743810 in TLR6 and overall susceptibility for leprosy were estimated in 114 cases and 456 controls. Following that, stratified analysis was performed. DNA was extracted from peripheral blood. Genotyping was performed using predesigned TaqMan probes. Results: The A/G genotype of rs5743810 behaved as a protective factor for the development of leprosy in the codominant (OR= 0.37; 95% CI = 016–0.86, *p* = 0.049) and over-dominant (OR = 0.38; 95% CI = 0.16–0.88, *p* = 0.019) inheritance models. The A/G and A/A genotypes behaved as a protective factor (OR = 0.39; 95% CI = 0.17–0.87, *p* = 0.016) in the dominant model. The SNVs rs5743618 and rs5743708 showed no association with any of the models. The CGG haplotype (rs5743618–rs5743708–rs5743810) behaved as a susceptibility factor for developing leprosy (OR = 1.86; 95% CI = 1.11–3.10, *p* = 0.019). The latter haplotype behaved as a susceptibility factor for leprosy development in women (OR = 2.39; 95% CI = 1.21–4.82, *p* = 0.013). Conclusions: The identified variants in the genes encoding TLRs, specifically rs5743810 in TLR6 and CGG (rs5743618–rs5743708–rs5743810) haplotypes, may somehow explain leprosy susceptibility in the studied population in a leprosy endemic region in Colombia.

## 1. Introduction

Leprosy is a chronic granulomatous disease caused by *Mycobacterium leprae* (*M. leprae*) which selectively invades macrophages, dendritic cells and Schwann cells [1,2,3,4]. In 2020, a total of 127.558 new cases of leprosy were reported worldwide, accounting for a prevalence of 16.7 per million population [5]. Although this pathology mainly affects the dermis and peripheral nerves, it can also spread to other areas such as the eyes, respiratory tract, muscle, bone and testes in some cases [1,6,7,8]. The clinical and immunological spectrum of leprosy is characterized by two major types: tuberculoid type (TT) and lepromatous type (LL), with transition stages including borderline tuberculoid (BT), borderline lepromatous (BL) and mid-borderline (BB) leprosy, depending on the proximity to either type [9]. The clinical manifestations and disease outcomes of these forms are closely related to the host innate immune response, which is highly associated with T cells [3,10,11].

Pattern recognition receptors (PRRs) are essential molecules that determine the activation of the immune response via the recognition of microorganisms by their antigens, called pathogen-associated molecular patterns (PAMPs) [12]. As PRRs, toll-like receptors (TLRs) play an important role in mediating the lipopeptide recognition of mycobacteria and the efficient activation of the immune response [1,4,7,11]. TLRs are type I transmembrane proteins characterized by an extracellular leucine-rich-repeat (LRR) domain, a transmembrane domain and a cytoplasmatic domain known as the toll/interleukin-1 receptor (TIR) [12,13]. The LRR domain identifies pathogens, while the TIR domain interacts with adapter proteins to stimulate the translocation of nuclear factor κB triggering the release of proinflammatory cytokines that determine the host immune response [12,13]. Thus, single nucleotide variants (SNVs) of these genes play an important role in determining the balance of proinflammatory and anti-inflammatory cytokines that modulate the immune response against pathogens and confer susceptibility or resistance to infectious and inflammatory diseases [7,8].

*M. leprae* antigens are mainly recognized by the TLR1/TLR2 and TLR2/TLR6 heterodimers. These receptors have been implicated in cell death, nerve damage and the invasion of Schwann cells by *M. leprae* [7,8,11]. Several studies have associated SNVs in these genes to resistance or susceptibility to leprosy and other immune diseases according to the analyzed population [12,13,14,15]. For example, Hong S.H et al. (2010) in a case–control study in the populations of New Delhi and Kolkata, India, found that the SNV rs5743618 in TLR1 was a protective factor against leprosy: OR = 0.27; 95% CI = 0.15–0.47, *p*-value = 3 × 10^−6^ and OR = 0.40; 95% CI = 0.20–0.83, *p*-value = 0.012, respectively [16]. Likewise, Johnson C.M et al. evidenced a protective effect of the G allele of this SNV against the development of leprosy in a population in Turkey (OR = 0.48 95% CI = 0.0.29–0.0.80, *p*-value < 0.05) [17]. However, this association was not validated in studies conducted in a population in China [18]. The rs5743708 SNV in TLR2 [13] and rs5743810 SNV in TLR6 have been associated with the immune response against mycobacteria [19,20,21]. Considering that the role of these variants in leprosy development or in immune response is dependent on the population studied, we conducted a case–control study to evaluate the association of the SNVs rs5743618 in TLR1, rs5743708 in TLR2 and rs5743810 in TLR6 in an endemic Colombian zone with age- and sex-stratified analysis. 

Colombia is the second most affected South American country in leprosy prevalence [5], with the presence of 3I and 4N *M. leprae* circulating genotypes. On the other hand, there are certain regions in the country such as Norte de Santander, with a high incidence of leprosy historically recorded, which moves us to study the genetic background of individuals; however, there are very few studies based on the genotypes of the Colombian *M. leprae* and fewer for each region.

## 2. Materials and Methods

### 2.1. Ethics Declaration

All procedures were conducted according to the Strengthening the Reporting of Genetic Association (STREGA) studies statement (Table S1) [22] This study was approved by the ethics committee of the Federico Lleras Acosta University Hospital Dermatology Center (assigned code 1DIS02-2Ñ; MinCiencias Code 212084368694). All participants signed an informed consent form, and all subjects were anonymized. The Helsinki Declaration ethical principles were followed, considering this a minimal risk research.

### 2.2. Study Population

Individuals included in this study were born and resided in Norte de Santander, a leprosy endemic region in Colombia. Cases were defined as individuals diagnosed with leprosy in accordance with the National Leprosy Program guidelines, identified using the National Public Health Surveillance System (SIVIGILA) records of Norte de Santander. All adult individuals diagnosed with leprosy per se, registered at any moment of their life, regardless of leprosy subtype, were included. All individuals were phoned to invite them to participate in the study. Patients were instructed to go to the health center nearest to their home, where they received an explanation about the study. Patients accepted the invitation to participate by signing an informed consent form. The control group included community members with no household or personal relationship to leprosy. Control group participants were also born and resided in Norte de Santander. They were recruited from primary care practices in different places such as universities, health centers and other entities, in different municipalities. They were individuals without a second or third degree of consanguinity with leprosy patients to minimize confusion or bias due to allelic enrichment. All subjects consented to participate by signing a control-group-specific informed consent form. The demographic, clinical and epidemiological data necessary for both cases and controls were recorded, which are archived together with the informed consent form that was signed by each of the participants in the study. The exclusion criteria for both cases and controls were not signing the informed consent or being a minor. For the controls, there was an exclusion criterion against those having a second or third degree of consanguinity with a case of leprosy or being a cohabitant of such a patient.

### 2.3. Study Design

A case–control study in individuals born and residing in Norte de Santander, Colombia, was conducted between 2020 and 2021 to identify the association between rs5743618 in TLR1, rs5743708 in TLR2 and rs5743810 in TLR6 SNVs and leprosy per se. The OpenEpi (Open-Source Epidemiologic Statistics for Public Health) Version 3.0.1 software using the Kelsey and Fleiss methods was used for sample size calculation [23]. The assumptions for calculation were extrapolated from the frequencies reported by dbSNP and Haploreg v4.1 for the rs5743618 and rs5743810 SNVs (allele frequencies between 0.38 and 0.40). We calculated a sample size of 570 individuals including 114 cases and 456 controls to produce a 95% confidence interval, (1-β) 80% power, a control/case ratio of 4:1, an expected allele frequency in controls of 38% and an odds ratio of 1.8. Sampling was carried out based on convenience in the municipalities of Norte de Santander. 

### 2.4. SNVs Selection

Variant selection was conducted by previous reports in different populations, and consequently the SNVs rs5743618 in TLR1 [12,24], rs5743708 in TLR2 [13] and rs5743810 in TLR6 [14,15] were chosen based on their association with infectious diseases and their role in *Mycobacterium leprae* antigen recognition.

### 2.5. DNA Extraction 

A blood sample was collected in EDTA vacutainer tubes (BD Vacutainer, Franklin Lakes, NJ, USA). Genomic DNA extraction from leukocytes, obtained from the total blood sample, was performed using the PureLink Genomic DNA extraction kit following the manufacturer’s recommendations. Quantification was performed using the NanoDrop 1000 Spectrophotometer (Thermo Scientific, Wilmington, DE, USA) and was subsequently frozen and stored at −20 °C until use. 

### 2.6. Genotyping

All three variants were genotyped by qPCR through predesigned and validated TaqMan probes: rs5743618 (C_175679112_10), rs5743708 (C_27860663_10) and rs5743810 (C_1180648_20). The PCR reaction was performed in a final volume of 10 μL: A DNA concentration of 2.5 ng/μL, 0.25 µL of the probe for each SNV and 5 μL of TaqMan Universal PCR Master Mix (Applied Biosystems, Austin, TX, USA) were used. The amplification conditions were: 1 cycle at 95 °C for 10 min, then 40 cycles at 95 °C for 15 s and at 60 °C for 1 min. All assays were performed in the StepOnePlus Real-Time PCR System. Genotypes were established by the presence or absence of the allele of interest, following the manufacturer’s recommendations. The verification of each person’s genotypes was conducted independently by two researchers to validate the results. In cases of non-concordance, the sample was amplified and sequenced again. 

### 2.7. Sequencing 

Randomly chosen samples were sequenced to verify the data found in qPCR genotyping. Five randomly chosen samples for each SNV were used in the sequencing process, using the following PCR conditions and reagents: Primers TLR1(ENSG00000174125): F-5′AGGGCTGGCCTGATTCTTAT-3′ and R-5′GCTCTTGCCAGGAACAAAGTTTC-3′; TLR2 (ENSG00000137462): F-5′TGATGCTGCCATTCTCATTC-3′ and R-5′CGCAGCTCTCAGATTTACCC-3′; TLR6 (ENSG00000174130): F-5′GAATGCAAAAACCCTTCACC-3′ and R-5′TGGGCCAAAGAAATTGAAAG-3′. The amplified segments were 392 bp, 157 bp and 245 bp, respectively. The amplification conditions were initial denaturation at 95 °C for 3 min, then 40 cycles at 95 °C for 10 s, at 60 °C for 30 s and at 72 °C for 30 s. PCR products were purified using the Monarch^®^ gel extraction kit from New England BioLabs Inc. (Ipswich, MA, USA).

The purified products were used as a template for forward and reverse sequencing by the Sanger method. The sequencing assay was conducted using the Big Dye Terminator v.3.1 cycle sequencing kit (4336917) and the Applied Biosystems, Austin, TX, USA, integrated systems for sequencing. The sequencing assay was performed using the GA3500 Applied Biosystems^®^ equipment. Sequences were analyzed using free BioEdit v7.2 software (Tom Hall; Ibis Biosciences, Carlsbad, CA, USA). Age and sex stratification, assignment to the case or control group and the detected allele were recorded in an Excel database for later analysis.

### 2.8. Statistical Analysis 

The Hardy–Weinberg equation was used to determine and compare the genotype and allele frequencies, and the Chi-square test was used to compare the proportions obtained in the HW equilibrium for each SNV analyzed in the case–control study. The odds ratio (OR) of exposure was calculated from the distribution of genotypes observed in cases and controls. The odds ratio and 95% confidence interval (CI) were computed to find the association between genotype and leprosy susceptibility using a linear regression model adjusted by age and sex for the detected genotype.

The association of each SNV was analyzed for codominant, dominant, over-dominant, recessive and log-additive models. A *p*-value < 0.05 was considered statistically significant. We decided to evaluate the association of each SNV and leprosy adjusted by sex, because differences determined by sex may influence the immune response. Haplotype frequencies were determined by the expectation-maximization (EM) algorithm, and the possible association of these haplotypes with leprosy was evaluated by the Chi-square test. Furthermore, the linkage disequilibrium (LD) algorithm analysis between SNVs and the permutation test for statistically significant results was performed using the “SNPStats” tool. The “SNPStats” software (https://www.snpstats.net/start.htm, accessed on 6 december 2022) was used for all of the analyses [25]. Linkage disequilibrium (LD) plots were generated using the Haploview v.4.2 program [26]. Haplotype blocks were identified using the «Solid Spine» (D’ > 0.8) algorithm implemented in the Haploview v.4.2 program [26].

## 3. Results

The mean age of cases was 57 (13–86) years and 37 (18–86) years for controls. The gender distribution was similar in cases and controls. The female/male ratio for cases was 56/58. By percentage, 51% and 50% were females for cases and controls, respectively.

All participants in this study feature Colombian ancestry and were born and residing in Norte de Santander. A total of 570 individuals were enrolled in the study (control group: n = 456 and case group: n = 114). No differences were found for age (χ2 = 0.92; *p* = 0.33) or sex (χ2 = 0.006; *p* = 0.93) distribution patterns in any of the evaluated groups. In total, 49% (controls: n = 227 and cases: n = 56) were women and 50.4% men (controls: n = 229 and cases: n = 58) 

### 3.1. Genotype and Allelic Distribution of SNVs

Allelic and genotypic frequencies for the rs5743618, rs5743708 and rs5743810 SNVs are described in Table 1. The genotypic distribution for the rs5743618, rs5743810 and rs5743708 SNVs was consistent with the HW equilibrium, for cases and controls. The G allele of the SNV rs5743708 was found to be fixed in the analyzed population.

The OR of exposure was evaluated through the different codominant, dominant, recessive, over-dominant and log-additive inheritance models to determine whether any of these variants could represent a resistance or susceptibility factor for the development of leprosy. These analyses were adjusted by age and sex. Neither of the two variants analyzed showed an association with disease, in data with or without adjusting by age or sex (Table S2 and Table 2). 

There was no association between leprosy susceptibility with any of the SNVs (rs5743618, rs5743708 and rs5743810) (Table S3) when analyzed by sub-groups in non-adjusted data, except for the AG genotype (rs5743810) (OR = 0.49; 95% CI = 0.24–0.99, *p* = 0.12) in the codominant model in men. However, when data were adjusted by age, we found the rs5743810 SNV in TLR6 entailed resistance to leprosy development in men (Table 3). The A/G genotype (rs5743810) is a resistance factor for leprosy development in the codominant model (OR = 0.37; 95% CI = 0.16–0.86, *p* = 0.049), as well as in the over-dominant model (OR = 0.38; 95% CI = 0.16–0.88 *p* = 0.019). The A/G-A/A genotypes proved to be a resistance factor (OR = 0.39; 95% CI = 0.17–0.87, *p* = 0.016) in the dominant model (Table 3). On the contrary, in women, there was no association between the rs5743618, rs5743708 and rs5743810 SNVs in any of the four inheritance models analyzed in data with or without adjusting by the age covariable (Table S4). The rs5743618 SNV showed no association in any of the analyzed models, in any sub-group (Table 3). 

### 3.2. Haplotype Analysis

**Figure 1:**
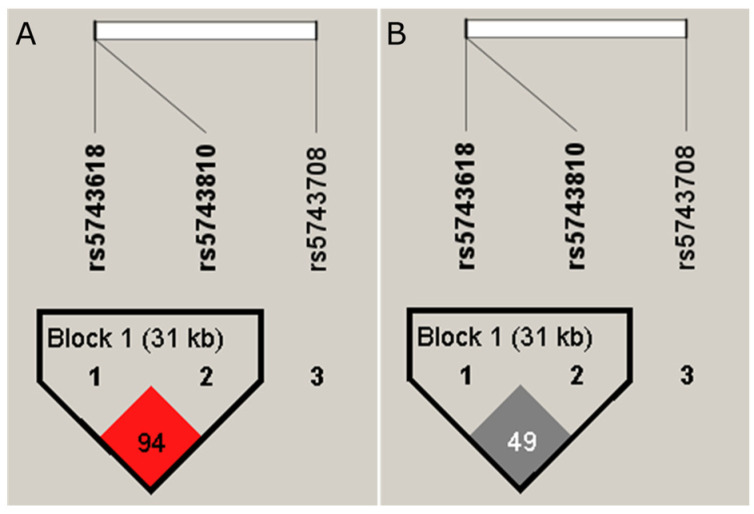
Schematic representation of the haplotype block formed by the SNVs rs5743618 (TLR1), rs5743810 (TLR6) and rs5743708 (TLR2). The ID of each SNV is taken from the reference sequences. The rs5743618 and rs5743810 SNVs form Block 1. (**A**) The LD (D′ = 0.94) among variants rs5743618 and rs5743810. (**B**) The value r2 = 0.9 among variants rs5743618 and rs5743810.

Haplotype analysis showed a moderate LD (linkage disequilibrium) between rs5743618–rs5743810 (r2 = 0.49, D’0.94) (Figure 1). The CGG haplotype (rs5743618–rs5743708–rs5743810) proved to be a susceptibility factor to leprosy (OR = 1.86; 95% CI = 1.11–3.10, *p* = 0.019) when adjusted by age and sex (Table 4). No association was observed even in non-adjusted data (Table S5). This haplotype was shown to be a susceptibility factor for leprosy in women (OR = 2.39; 95% CI = 1.21–4.72, *p* = 0.0013) (Table 5), as was the AGA haplotype (rs5743618–rs5743708–rs5743810) (OR = 6.92; 95% CI = 1.08–44.25, *p* = 0.042) (Table 5).

## 4. Discussion

A case–control study was conducted in a leprosy endemic region of Colombia (Norte de Santander) to investigate the potential association between single nucleotide variants of the TLR1, TLR2 and TLR6 genes and the development of leprosy. This investigation was prompted by previous findings indicating that SNVs in these genes are associated with leprosy resistance or susceptibility in different populations [13,14,24].

Among the identified antigens of *M. leprae*, thirty-one lipoproteins including lipomannan, lipoarabinomannan, phosphatidylinositol dimannoside and a 19 kDa lipoprotein, exhibit a binding affinity for the TLR2/1 heterodimer [13,24]. The TLR2/1 heterodimer has been implicated in triggering cellular activation against *M. leprae* [2], whereas the TLR6-mediated response has been associated with Virchow cell formation, a feature within lepromatous lesions [7,8,15]. Furthermore, it has been postulated that the TLR2 homodimer may participate in the response to *M. leprae* [27]. Nevertheless, the structure of the TLR2 homodimer has not been described [28].

The rs5743618 variant in TLR1 is one of the most studied. Located at position chr4:38797027, this variant involves a substitution of adenine for cytosine (A1805C), resulting in a a change from isoleucine to serine at Position 602 (I602S) in the protein [29]. The overall frequency of the rs5743618 SNV is 40.423% and 59.577% for the A and C alleles, respectively. The distribution of these alleles varies according to ancestry. Among Caucasians, the 602I allele comprises 25%, while the 602S allele represents about 75%. Among Afro-American populations, the frequency has been estimated at ~75% for the 602I allele and for the 25% 602S allele. Similarly, within Hispanic, Turkish and Nepalese populations, the frequencies are ~70/30, 57/43 and ~94/6% for the 602I/602S alleles, respectively. In Western Asian individuals, the 602I allele frequency is >99%, while the 602S allele appears to be absent [29,30].

In this study, we found that the frequencies of the 602I/602S alleles in the analyzed population were 75%/25% in cases and 72%/29% in controls, which closely resemble the findings observed in Afro-American and Hispanic populations [29,30]. Nevertheless, these frequencies are not related with previous ancestry studies conducted in the same populations. Those earlier studies reported a prevalence of 7.4% among individuals of African descent, 57.99% among those of European descent and 34.97% among individuals with Native American ancestry [31]. Remarkably, we observed a higher frequency of the African related allele (602I). Furthermore, our results indicate that the 602I allele is prevalent in the population compared to the 602S allele, which shows a higher frequency among those of European and European American descent [16].

The rs5743618 SNV was found in HW equilibrium in the analyzed population. In addition, the presence of the C allele (rs5743618) in the CGG haplotype (rs5743618–rs5743708–rs5743810) (Table 5) suggests a susceptibility factor for the development of leprosy, so it is possible that the selection of the 602I allele (1805A) along with the genetic background of this population determine the susceptibility to leprosy.

The levels of the TLR1 receptor on the membrane of peripheral blood monocytes are low in homozygous individuals for the S allele, compared with individuals who are heterozygous or homozygous for the 602I allele [17,32]. Likewise, low TLR1 activity results in lower FNT-α activation, which has been associated with a protective effect against leprosy [17,32]. In the context of our study, we did not identify differences in the presence of the C/C genotype between cases (7%) and controls (10%). However, it is plausible that the relatively low frequency of this allele (602S; C) in combination with other genetic variants involved in the immune response could play an important role in contributing to the endemic nature of the disease in this population.

It should be noted that the present study did not find evidence to support the hypothesis that the presence of the allele C could be a protective factor in the analyzed population in the different inheritance models (Table 2 and Table 3), nor did we find any association in the sex-stratified population. Previous studies have suggested that the presence of the 602S variant confers resistance to leprosy, as evidenced by studies conducted in New Delhi (OR = 0.27; 95% CI = 0.15−0.47), Kolkata (OR = 0.40; 95% CI = 0.20–0.83), Kumbakonam family (OR = 0.61, 95% CI =0.35–1.09) and Turkey (OR = 0.37; 95% CI = 0.26–0.51) [22] populations. Furthermore, it has been demonstrated that the inclusion of the 602S variant in the haplotype analysis significantly affects the observed association in this region against leprosy susceptibility. These results show that susceptibility to leprosy in this population is not exclusively determined by the presence of the 602I allele.

On the other hand, the rs5743708 (2258G>A) variant leads to an arginine-to-glutamine substitution at Residue 753 (Arg753Gln) [33] in the TLR2 gene. This variant has been the least studied in leprosy. Nonetheless, an association with TLR2 malfunction has been found [34]. This variant has a global frequency of 97.373% for the G allele and 2.627% for the A allele [35]. This is consistent with the present study findings, in which we found a frequency of 99.9% for the G allele, showing no association with leprosy development. Given that the TLR2 gene encompasses a broad spectrum of pathogen-associated molecular pattern (PAMP) recognition receptors, including triacylated oligopeptides and diacylated lipopeptides [36], it has been widely studied in tuberculosis [19]. Notably, the rs121917864 (c.2029C>T, pArg677Trp) variant of the TLR2 gene has been associated with a decrease in the immune response to *M. leprae*. A study by Bochud P.Y et al. 2003, reported the important role played by the TLR2 gene in the response to *M. leprae* [37]. In this study, HEK29 cells transfected with this variant (p. Arg677Trp) led to the impairment of NF-kB pathway activation [37], providing clear evidence of the compromised immune response to mycobacteria caused by TLR2 variants.

The rs5743810 (745 G>A) variant within the TLR6 gene is located on an exonic region of Chromosome 4. In the analyzed population, this variant exhibited a frequency of 82% for the G allele and 18% for the A allele, similar to the global frequency data (A allele: 38.13% and G allele: 61.8699) [38]. The rs5743810 variant leads to a proline-to-serine change at Position 249 (S249P) in the receptor’s extracellular domain. It has been demonstrated that in peripheral blood monocytes, the G-rs5743810 allele has a better NF-kB signal activation than that found with the T-rs5743810 allele [39]. Additionally, this variant has been associated with altered IL6 levels in response to lipopeptides from *Mycobacterium tuberculosis* lysates [14]. This SNV has also been associated with the response against *Mycobacterium leprae*. It has been proposed that the innate immune response in infected Schwann cells depends on lipid droplets and TLR2/TLR6 heterodimer signaling, driving apoptosis and possibly contributing to nerve damage in this disease [15,40]. In individuals with tuberculosis, the G/A (TLR6-rs5743810) and G/T (TLR10-rs11096957) genotypes have a significant association with a higher susceptibility to developing pulmonary tuberculosis (OR = 2.48, 95% CI 1.62–3.85) [41].

The association analysis conducted in the present study established a link between the rs5743810 variant and protective effects against leprosy development in males, across dominant, codominant, over-dominant and additive models (Table 3). Finally, the haplotype analysis showed the presence of one haplotype (618G, 810G, 810C) as a susceptibility to the leprosy factor in this population (Table 4). According to our results, the association of this SNV with leprosy susceptibility may explain the higher incidence of this disease within the Colombian region under study [42].

The recognition of variants in these TLR-encoding genes helps to explain the different degrees of susceptibility in Colombian population, as well as the outcome this entails due to the immune response variability of the disease. The evaluated SNVs show that the rs5743618 variant in the TLR1 gene, in combination with the rs5743708 SNV in the TLR2 gene and the rs5743810 SNV in the TLR6 gene, are related with the susceptibility to leprosy in this population. This effect can be diminished by heterodimerization with TLR2. Additionally, the SNV rs5743810 seems to function as a protective factor against leprosy, specifically in males. The presence of the G allele in the population for the rs5743708 SNV in the TLR2 gene may play an important role or mediate PAMP recognition of *Mycobacterium leprae.* This contributes to explain the endemic nature of the disease in this population, as each heterodimer recognizes different PAMPs; for example, the TLR1/2 heterodimer recognizes triacylated lipopeptides, while the TLR2/6 recognizes diacylated lipopeptides [40].

## 5. Conclusions

In conclusion, this study is the first to show that the rs5743810 variant of the TLR6 gene is associated with resistance to the development of leprosy in men from the analyzed Colombian population, which is differs from the results reported for the widely studied Asian population. Additionally, the CGG (rs5743618–rs5743708–rs5743810) haplotype is a leprosy susceptibility factor that could be used for the application of public health measures recently designed by the WHO, such as chemoprophylaxis. It is important to evaluate other variants of these and other genes in this population in order to identify the different SNVs associated with leprosy susceptibility that may explain the prevalence of this disease in this population.

## 6. Study Limitations

The results obtained in this study demonstrate different limits of interpretation. For example, only one SNV was analyzed for each gene, which leads to difficulty in observing a higher statistical power in the association found between gene variants and their role in leprosy susceptibility. Despite the inclusion of a substantial number of people diagnosed with leprosy (114), it is necessary to increase the sample size to make conclusions on the role of these SNVs in the population of this Colombian region. It is important to note that due to the genetic heterogeneity of the Colombian population, the replication of this study is needed in other leprosy-endemic regions of Colombia.

## Figures and Tables

**Table 1:** Allelic and genotypic frequencies of the rs5743618, rs5743708 and rs5743810 SNVs.

Gen/SNP	Allele/ Genotype	Cases		Controls		χ2	*p*-Value
		(n = 114)		(n = 456)			
		N	Frequency	n	Frequency		
TLR1 (rs5743618)	A	166	0.73	647	0.71	0.309	0.5778
	C	62	0.27	265	0.29		
	A/A	61	0.54	231	0.51	0.314	0.8543 *
	A/C	44	0.39	185	0.41		
	C/C	9	0.08	40	0.09		
TLR2 (rs5743708)	G	227	1	909	1	0.062	0.8022 *
	A	1	0	3	0		
	G/A	1	0.01	3	0.01	0.062	0.8019
	G/G	113	0.99	453	0.99		
TLR6 (rs5743810)	G	194	0.85	738	0.81	2.12	0.1451
	A	34	0.15	174	0.19		
	A/A	3	0.03	14	0.03	2.57	0.2760
	G/A	28	0.25	146	0.32		
	G/G	83	0.73	296	0.65		

* Chi-square: Fisher’s exact test.

**Table 2:** Genotype distribution for the TLR1 and TLR6 SNVs in leprosy cases and controls in inheritance models (n = 570).

Gene/SNV	Model	Genotype	Cases	Controls	OR (95% CI)	*p*-Value *
			n (Frequency)	n (Frequency)		
TLR1 rs5743618	Codominant	A/A	61	231 (50.7%)	1	0.99
			−53.50%			
		C/A	44	185 (40.6%)	0.98 (0.60–1.59)	
			−38.60%			
		C/C	9	40	0.95 (0.40–2.25)	
			−7.90%	−8.80%		
	Dominant	A/A	61 (53.5%)	231 (50.7%)	1	0.91
		C/A-C/C	53	225 (49.3%)	0.97 (0.61–1.54)	
			−46.50%			
	Recessive	A/A-C/A	105 (92.1%)	416 (91.2%)	1	0.92
		C/C	9	40 (8.8%)	0.96 (0.41–2.21)	
			−7.90%			
	Over-dominant	A/A-C/C	70 (61.4%)	271 (59.4%)	1	0.95
		C/A	44 (38.6%)	185 (40.6%)	0.99 (0.62–1.58)	
	Log-additive	---	---	---	0.98 (0.68–1.40)	0.89
TLR6 rs5743810	Codominant	G/G	83 (72.8%)	296 (64.9%)	1	0.18
		A/G	28 (24.6%)	146 (32%)	0.61 (0.6–1.04)	
		A/A	3 (2.6%)	14 (3.1%)	0.75 (0.18–3.04)	
	Dominant	G/G	83 (72.8%)	296 (64.9%)	1	0.066
		A/G-A/A	31 (27.2%)	160 (35.1%)	0.63 (0.38–1.04)	
	Recessive	G/G-A/G	111 (97.4%)	442 (96.9%)	1	0.83
		A/A	3 (2.6%)	14 (3.1%)	0.86 (0.21–3.47)	
	Over-dominant	G/G-A/A	86 (75.4%)	310 (68%)	1	0.07
		A/G	28 (24.6%)	146 (32%)	0.62 (0.37–1.05)	
	Log-additive	---	---	1.46 (0.93–2.29)	0.68 (0.44–1.08)	0.092

* Data adjusted by age and sex.

**Table 3:** Genotype distribution for the rs5743618 (TLR1) and rs5743810 (TLR6) SNVs in the case–control study in the male subgroup. (n = 287).

Gene/SNV	Model	Genotype	Cases n (Frequency)	Controls n (Frequency)	OR (95% CI)	*p*-Value *
TLR1 rs5743618	Codominant	A/A	32 (55.2%)	107 (46.7%)	1	0.46
		C/A	22 (37.9%)	100 (43.7%)	0.68 (0.33–1.41)	
		C/C	4 (6.9%)	22 (9.6%)	0.54 (0.14–2.06)	
	Dominant	A/A	32 (55.2%)	107 (46.7%)	1	0.23
		C/A-C/C	26 (44.8%)	122 (53.3%)	0.66 (0.33–1.31)	
	Recessive	A/A-C/A	54 (93.1%)	207 (90.4%)	1	0.5
		C/C	4 (6.9%)	22 (9.6%)	0.65 (0.18–2.35)	
	Over-dominant	A/A-C/C	36 (62.1%)	129 (56.3%)	1	0.4
		C/A	22 (37.9%)	100 (43.7%)	0.74 (0.37–1.50)	
	Log-additive	---	---	---	0.71 (0.41–1.23)	0.22
TLR6 rs5743810	Codominant	G/G	45 (77.6%)	147 (64.2%)	1	0.049
		A/G	11 (19%)	74 (32.3%)	0.37 (0.16–0.86)	
		A/A	2 (3.5%)	8 (3.5%)	0.56 (0.09–3.52)	
	Dominant	G/G	45 (77.6%)	147 (64.2%)	1	0.016
		A/G-A/A	13 (22.4%)	82 (35.8%)	0.39 (0.17–0.87)	
	Recessive	G/G-A/G	56 (96.5%)	221 (96.5%)	1	0.74
		A/A	2 (3.5%)	8 (3.5%)	0.74 (0.12–4.53)	
	Over-dominant	G/G-A/A	47 (81%)	155 (67.7%)	1	0.019
		A/G	11 (19%)	74 (32.3%)	0.38 (0.16–0.88)	
	Log-additive	---	---	---	0.49 (0.25–0.97)	0.032

* Data adjusted by age.

**Table 4:** Haplotype association with leprosy in the case and control groups (n = 570).

Haplotype	rs5743618	rs5743708	rs5743810	Frequency	OR (95% CI)	*p*-Value *
1	A	G	G	0.7044	1	---
2	C	G	A	0.1756	0.65 (0.40–1.06)	*0.082*
3	**C**	**G**	**G**	0.1096	1.86 (1.11–3.10)	0.019

* Age- and sex-adjusted data.

**Table 5:** Haplotype association with leprosy in a case–control study in the female and male subgroups.

	Female Subgroup, n = 283.					
Haplotype	rs5743618	rs5743708	rs5743810	Frequency	OR (95% CI)	*p*-Value *
1	A	G	G	0.71	1	---
2	C	G	A	0.17	0.81 (0.42–1.59)	0.55
3	C	G	G	0.10	2.39 (1.21–4.72)	0.013
4	A	rs5743708 G	A	0.01	6.92 (1.08–44.25)	0.042
	Global haplotype association *p*-value: 0.017					
	**Male Subgroup, n = 287**.					
Haplotype	rs5743618	rs5743708	rs5743810	Frequency	OR (95% CI)	*p*-value *
1	A	G	G	0.6928	1	---
2	C	G	A	0.181	0.51 (0.25–1.01)	0.056
3	C	G	G	0.1207	1.32 (0.59–2.93)	0.5
	Global haplotype association *p*-value: 0.15					

* Age-adjusted data.

## Data Availability

All the information is in this paper.

## Supplementary Materials

The following supporting information can be downloaded at: https://www.mdpi.com/article/10.3390/tropicalmed8100473/s1, Table S1. Strengthening the Reporting of Genetic Association (STREGA) studies reporting recommendations, extended from the STROBE statement; Table S2. Distribution of SNV genotypes of TLR1 and TLR6 in leprosy cases and controls in inheritance models; Table S3. Genotype distribution of TLR1 and TLR6 in leprosy cases and controls in models of inheritance in the male subgroup; Table S4. Genotype distribution of TLR1 and TLR6 in leprosy cases and controls in inheritance models in the female subgroup; Table S5. Haplotype association with leprosy in the case–control group.
