# Supplementary material for: Single Nucleotide Variants in the TLR1, TLR2 and TLR6 Genes: A Case–Control Study in a Colombian Population
Source: Trop Med Infect Dis. 2023 Oct 16;8(10):473. doi: 10.3390/tropicalmed8100473 (PMC10610572; doi:10.3390/tropicalmed8100473)
Supplement: Supplementary file 1 [file tropicalmed-08-00473-s001.zip › Table S2.pdf]

Table S2. Distribution of SNV genotypes of TLR1 and TLR6 in leprosy cases and controls in inheritance models. (n=570, non-adjusted data).

Gene/SNV	Model	Genotype	Cases n (frequencies)	Controls n (frequencies)	OR (95%CI)	*P-value*
TLR1 rs5743618	**Codominant**	A/A	61 (53.5%)	231 (50.7%)	1	0,85
		C/A	44 (38.6%)	185 (40.6%)	0.90 (0.58-1.39)	
		C/C	9 (7.9%)	40 (8.8%)	0.85 (0.39-1.85)	
	**Dominant**	A/A	61 (53.5%)	231 (50.7%)	1	0,59
		C/A-C/C	53 (46.5%)	225 (49.3%)	0.89 (0.59-1.35)	
	**Recessive**	A/A-C/A	105 (92.1%)	416 (91.2%)	1	0,76
		C/C	9 (7.9%)	40 (8.8%)	0.89 (0.42-1.89))	
	**Overdominant**	A/A-C/C	70 (61.4%)	271 (59.4%)	1	0,7
		C/A	44 (38.6%)	185 (40.6%)	0.92 (0.60-1.40)	
	**Log-additive**	---	---	---	0.91 (0.66-1.26)	0,58
TLR6 rs5743810	**Codominant**	G/G	83 (72.8%)	296 (64.9%)	1	0,27
		A/G	28 (24.6%)	146 (32%)	0.68 (0.43-1.10)	
		A/A	3 (2.6%)	14 (3.1%)	0.76 (0.21-2.72)	
	**Dominant**	G/G	83 (72.8%)	296 (64.9%)	1	0,11

		A/G-A/A	31 (27.2%)	160 (35.1%)	0.69 (0.44-1.09)	
	**Recessive**	G/G-A/G	111 (97.4%)	442 (96.9%)	1	0,8
		A/A	3 (2.6%)	14 (3.1%)	0.85 (0.24-3.02)	
	**Overdominant**	G/G-A/A	86 (75.4%)	310 (68%)	1	0,12
		A/G	28 (24.6%)	146 (32%)	0.69 (0.43-1.11)	
	**Log-additive**	---	---	---	0.74 (0.49-1.11)	*0,13*
