# Supplementary material for: Single Nucleotide Variants in the TLR1, TLR2 and TLR6 Genes: A Case–Control Study in a Colombian Population
Source: Trop Med Infect Dis. 2023 Oct 16;8(10):473. doi: 10.3390/tropicalmed8100473 (PMC10610572; doi:10.3390/tropicalmed8100473)
Supplement: Supplementary file 1 [file tropicalmed-08-00473-s001.zip › Table S3.pdf]

Table S3. Genotypes distribution of TLR1 and TLR6 in leprosy cases and controls in models of inheritance in the male subgroup. (n=287, non-adjusted data).

Gene/SNV	Model	Genotype	Cases n (frequency)	Controls n (frequency)	OR (95%CI)	*P-value*
TLR1 rs5743618	Codominant	A/A	32 (55.2%)	107 (46.7%)	1	0.49
		C/A	22 (37.9%)	100 (43.7%)	0.74 (0.40-1.35)	
		C/C	4 (6.9%)	22 (9.6%)	0.61 (0.20-1.89)	
	Dominant	A/A	32 (55.2%)	107 (46.7%)	1	0.25
		C/A-C/C	26 (44.8%)	122 (53.3%)	0.71 (0.40-1.27)	
	Recessive	A/A-C/A	54 (93.1%)	207 (90.4%)	1	0.51
		C/C	4 (6.9%)	22 (9.6%)	0.70 (0.23-2.11)	
	Overdominant	A/A-C/C	36 (62.1%)	129 (56.3%)	1	0.43
		C/A	22 (37.9%)	100 (43.7%)	0.79 (0.44-1.42)	
	Log-additive	---	---	---	0.76 (0.48-1.21)	0.24
TLR6 rs5743810	Codominant	G/G	45 (77.6%)	147 (64.2%)	1	0.12
		A/G	11 (19%)	74 (32.3%)	**0.49 (0.24-0.99)**	
		A/A	2 (3.5%)	8 (3.5%)	0.82 (0.17-3.99)	
	Dominant	G/G	45 (77.6%)	147 (64.2%)	1	0.047
		A/G-A/A	13 (22.4%)	82 (35.8%)	0.52 (0.26-1.02)	
	Recessive	G/G-A/G	56 (96.5%)	221 (96.5%)	1	0.99
		A/A	2 (3.5%)	8 (3.5%)	0.99 (0.20-4.78)	

	Overdominant	G/G-A/A	47 (81%)	155 (67.7%)	1	0.04
		A/G	11 (19%)	74 (32.3%)	0.49 (0.24-1.00)	
	Log-additive	---	---	---	0.61 (0.34-1.10)	0.085
