# Supplementary material for: Single Nucleotide Variants in the TLR1, TLR2 and TLR6 Genes: A Case–Control Study in a Colombian Population
Source: Trop Med Infect Dis. 2023 Oct 16;8(10):473. doi: 10.3390/tropicalmed8100473 (PMC10610572; doi:10.3390/tropicalmed8100473)
Supplement: Supplementary file 1 [file tropicalmed-08-00473-s001.zip › Table S4.pdf]

Table S4. Genotypes distribution of TLR1 and TLR6 in leprosy cases and controls in inheritance models in the female subgroup. (n=283).

Gene/SNV	Model	Genotype	Cases n (Frecuency)	Controls n (Frecuency)	OR (95%CI)	*P-value*
TLR1 rs5743618	Codominant	A/A	29 (51.8%)	124 (54.6%)	1	0. 92
		C/A	22 (39.3%)	85 (37.4%)	1.11 (0.60-2.06)	
		C/C	5 (8.9%)	18 (7.9%)	1.19 (0.41-3.46)	
	Dominant	A/A	29 (51.8%)	124 (54.6%)	1	0.7
		C/A-C/C	27 (48.2%)	103 (45.4%)	1.12 (0.62-2.01)	
	Recessive	A/A-C/A	51 (91.1%)	209 (92.1%)	1	0.81
		C/C	5 (8.9%)	18 (7.9%)	1.14 (0.40-3.21)	
	Overdominant	A/A-C/C	34 (60.7%)	142 (62.6%)	1	0. 8
		C/A	22 (39.3%)	85 (37.4%)	1.08 (0.59-1.97)	
	Log-additive	---	---	---	1.10 (0.70-1.72)	0.61
TLR6 rs5743810	Codominant	G/G	38 (67.9%)	149 (65.6%)	1	0.9
		A/G	17 (30.4%)	72 (31.7%)	0.93 (0.49-1.75)	
		A/A	1 (1.8%)	6 (2.6%)	0.65 (0.08-5.59)	
	Dominant	G/G	38 (67.9%)	149 (65.6%)	1	0.75
		A/G-A/A	18 (32.1%)	78 (34.4%)	0.90 (0.48-1.69)	
	Recessive	G/G-A/G	55 (98.2%)	221 (97.4%)	1	0.7
		A/A	1 (1.8%)	6 (2.6%)	0.67 (0.08-5.68)	

	Overdominant	G/G-A/A	39 (69.6%)	155 (68.3%)	1	084
		A/G	17 (30.4%)	72 (31.7%)	0.94 (0.50-1.77)	
	Log-additive	---	---	---	0.89 (0.51-1.57)	0.7
