# Supplementary material for: Single Nucleotide Variants in the TLR1, TLR2 and TLR6 Genes: A Case–Control Study in a Colombian Population
Source: Trop Med Infect Dis. 2023 Oct 16;8(10):473. doi: 10.3390/tropicalmed8100473 (PMC10610572; doi:10.3390/tropicalmed8100473)
Supplement: Supplementary file 1 [file tropicalmed-08-00473-s001.zip › Table S5.pdf]

Table S5. Haplotype association with leprosy in case-control group (n=570, non-adjusted data).

**Haplotype**	**rs5743618**	**rs5743708**	**rs5743810**	**OR (95% CI)**	***p-value***	**Haplotype**
1	A	G	G	0.7044	1	---
2	C	G	A	0.1756	0.68 (0.44 - 1.05)	0.084
3	C	G	G	0.1096	1.44 (0.92 - 2.25)	0.11
**Global haplotype association** ***p-value: 0.018***						
